# A nomogram to predict residual cavity formation after thoracoscopic decortication in chronic tuberculous empyema

**DOI:** 10.1093/icvts/ivac011

**Published:** 2022-02-11

**Authors:** Pengfei Zhu, Xudong Xu, Bo Ye, Guocan Yu, Likui Fang, Wenfeng Yu, Fangming Zhong, Xiaowei Qiu, Xin Yang

**Affiliations:** 1 Department of Thoracic Surgery, Affiliated Hangzhou Chest Hospital, Zhejiang University School of Medicine, Hangzhou, China 310003; 2 Department of Radiology, Affiliated Hangzhou Chest Hospital, Zhejiang University School of Medicine, Hangzhou, China. 310003

**Keywords:** chronic tuberculous empyema, risk factors, nomogram, decortication

## Abstract

**OBJECTIVES:**

The goal of this study was to develop and validate a nomogram for predicting residual cavity formation after video-assisted thoracoscopic decortication in patients with chronic tuberculous empyema (CTE).

**METHODS:**

We retrospectively analysed patients who were diagnosed and treated for CTE at our hospital from January 2017 to December 2020. We used univariable and binary logistic regression analyses to identify independent risk factors. A predictive nomogram was developed and validated for predicting the risk of residual cavity formation after video-assisted thoracoscopic decortication in patients with CTE. The receiver operating characteristic (ROC) was used to evaluate the nomogram.

**RESULTS:**

Data from 103 patients were analysed. The contact area between the lung and empyema (P = 0.001, odds ratio [OR] 1.017, 95% confidence interval [CI] 1.007–1.028), calcification (P = 0.004, OR 0.12, 95% CI 0.029–0.501) and thickness of the pleura (P = 0.02, OR 1.315, 95% CI 1.045–1.654) were risk factors for residual cavity formation after video-assisted thoracoscopic decortication. A 50% residual cavity formation rate was used as the cut-off to validate the nomogram model. The area under the ROC curve for the nomogram was 0.891 (95% CI, 0.82–0.963). The sensitivity and specificity of the nomogram were 86.67% and 82.19%, respectively. The calibration curve indicated good consistency between the predicted and actual risks.

**CONCLUSIONS:**

The preliminary nomogram could contribute to preventing postoperative residual cavity formation and making appropriate surgical decisions.

**Figure:**
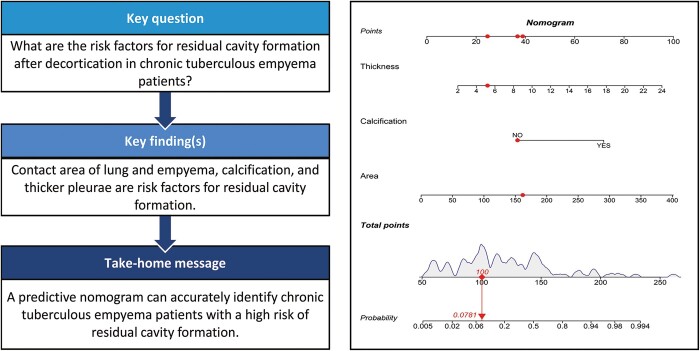

## INTRODUCTION

Tuberculosis (TB) is one of the most common causes of death from a single infectious pathogen, especially in developing countries [1]. Tuberculous empyema [2] develops due to rupture of a subpleural pulmonary lesion or from nodal, lymphatic or haematogenous spread of the primary pulmonary disease [3]. Tuberculous empyema cannot be treated with antituberculosis drugs alone; surgical evacuation of the pus is required [4]. Video-assisted thoracoscopic surgery (VATS) is used extensively for the decortication of tuberculous empyema with good results at multiple centres [5–7]. The most important requirement in empyema decortication is allowing the lungs to fill up the pleural cavity completely. Any remaining space in the cavity inevitably causes recurrence of empyema. Once empyema forms again, a second operation may be necessary. Repeat decortication is difficult and is best avoided; thus, the first decortication attempt is crucial [8].

Accurate prediction of the probability of residual cavity formation based on clinical characteristics and preoperative imaging features may help clinicians take necessary preventive measures in advance to improve the success rate of the operation; however, few articles describe the factors that influence postoperative residual cavity formation. In this study, our goal was to identify the predictors of postoperative cavity formation using univariable and multivariable analyses, develop a nomogram model to predict the probability of residual cavity formation and validate the nomogram.

## METHODS

### Ethical statement

The study protocol was approved by the institutional review board of the Affiliated Hangzhou Chest Hospital, Zhejiang University School of Medicine on 15 June 2021. Written patient informed consent was obtained.

### Patient selection

Chronic tuberculous empyema (CTE) was defined as tuberculous empyema lasting for >6 weeks. A diagnosis of CTE was made based on the following diagnostic criteria: (1) positive acid-fast bacilli staining of pleural effusion or tissue specimen; (2) positive mycobacterium TB pleural effusion or tissue culture result; or (3) a pleural specimen exhibiting granulomatous inflammation with caseous necrosis or positive acid-fast bacilli after examination of a histopathological tissue section (ruling out nontuberculous mycobacterial infection). All patients included in the study were diagnosed with CTE and underwent decortication using VATS at our hospital from January 2017 to December 2020. Patients were excluded from the study if they had a pneumonectomy, TB of the chest wall or a tumour or if the course of the disease was <6 weeks.

### Data collection

Demographic and comorbidity data, including sex, age, clinical characteristics, mean arterial pressure, heart rate, white blood cell count, creatinine, albumin, C-reactive protein, forced expiratory volume in 1 s (FEV1), left ventricular ejection fraction (LVEF; measured by echocardiogram), TB treatment time, lactate, symptom duration, duration of the operation, the extubation time, the postoperative hospital stay, in-hospital deaths, 90-day deaths and pulmonary tuberculosis complications, were collected. Moreover, radiological data, including the contact area between the lung and the empyema, the thickness of the pleura and pleural calcification, were collected.

Computed tomography (CT) graphs were visualized using ImageJ 1.45 s software (http://imagej.net/ImageJ). The segmented line tool in ImageJ 1.45 s software was selected to manually anchor the length of the contact surface between the empyema and the lung. The Fit Spline tool (under the edit selection menu) was selected to process the anchored lines. Under the Analyze menu, Measure was selected to calculate the length of the CT plane. The contact area of each CT layer was defined as the length of the contact surface between the empyema and the lung multiplied by the thickness of the CT slices. The contact area between the lung and the empyema was defined as the sum of the contact area of each CT layer (Supplementary Fig. 1).

On the CT scan, pleural calcification was defined as dot-like, nodule-like, linear or high- density irregular foci of 80–300 Hounsfield units located on the pleura. Pleural thickness was measured using axial CT images, with the empyema divided into 3 levels (upper, middle and lower). The maximum pleural thickness perpendicular to the chest wall or mediastinum was measured using axial imaging at each of the 3 levels and then averaged.

Two experienced observers—an imaging physician and a thoracic surgeon—who were blinded to the clinical information reviewed all images to identify the imaging markers. Discrepancies regarding the presence of the markers were resolved through consensus.

### Surgery and residual cavity

The indications for decortication included trapped lung due to thickened pleural cortex, empyema with or without bronchopleural fistula, disease progression or relapse despite antituberculosis therapy and symptomatic bronchiectasis. Antituberculosis therapy was recommended for at least 6 weeks before decortication. For patients with CTE, we usually try to perform a thoracoscopic operation. However, if complete decortication would be difficult to be achieved using VATS, we choose thoracotomy.

After induction of general anaesthesia and double-lumen endotracheal intubation, patients were placed in the lateral position with the arm adducted and flexed at the shoulder and elbow. A standard 10-mm port was placed in the seventh intercostal space on the midaxillary line. The affected lung was allowed to collapse and the opposite lung was ventilated. Following insertion of a standard 30° laparoscope (Storz, Tuttlingen, Germany) and inspection of the pleural space, one or two 5-mm additional ports were inserted in suitable locations. Prior to port insertion, port sites were infiltrated and appropriate intercostal nerves were blocked with 0.5% bupivacaine to a maximum dose of 1 mg/kg body weight. Thickened parietal pleurae were resected by detaching them from the endothoracic fascia by extrapleural dissection. The fibrinous peel constricting the visceral pleura was released carefully to avoid air leakage; the diaphragmatic pleura was released as completely as possible. All fluid was aspirated and sent for bacteriological and cytological examination. Fibrous material and dense parietal and visceral rinds were removed and sent for histological examination. Lung expansion was assessed via manual ventilation. All significant air leaks were meticulously closed using polyglactin absorbable sutures (Vicryl). The thoracic cavity was thoroughly irrigated with a mixture of normal saline, povidone-iodine and hydrogen peroxide until all flakes were cleared. One chest tube was inserted. The chest tube was removed when there was no active air leak and the volume of drainage was less than 100 ml/day. The patient was discharged when he/she had no or mild symptoms.

Chest roentgenograms before discharge from the hospital and at 1, 3 and 6 months postoperatively were compared with the preoperative roentgenograms. The radiological improvement was calculated using a five-point percentage scale [9]. Complete (100%) to moderate (75%) improvement in lung re-expansion at any time during the follow-up period was considered satisfactory. Residual cavity formation was defined as lung re-expansion of less than 75% 6 months postoperatively.

### Statistical analysis

Patients were assigned to residual cavity formation or no residual cavity formation groups. The measurement data and numeric data were analysed statistically with *t*-tests and χ^2^ tests, respectively. Clinical characteristics with P values <0.1 were included in the multivariable analysis using binary logistic regression to identify the independent risk factors predicting residual cavity formation. P values, odds ratios (OR) and 95% confidence intervals (CI) are used to describe the risk factors for residual cavity formation. Based on the multivariable analysis, a nomogram was established using the rms package in R. The receiver operating characteristic (ROC) curve was applied to evaluate model discrimination, and the area under the curve (AUC) value is shown. A higher AUC indicates a higher prediction power. Cut-off values were determined based on the ROC curve. A calibration curve was used to assess the calibration of the nomogram. All statistical analyses were performed using R software (MathSoft, Cambridge, MA, USA) and SPSS (version 26, IBM, USA). Statistical significance was set at P value < 0.05 (two-sided).

## RESULTS

### Patient characteristics

**Figure 1:**
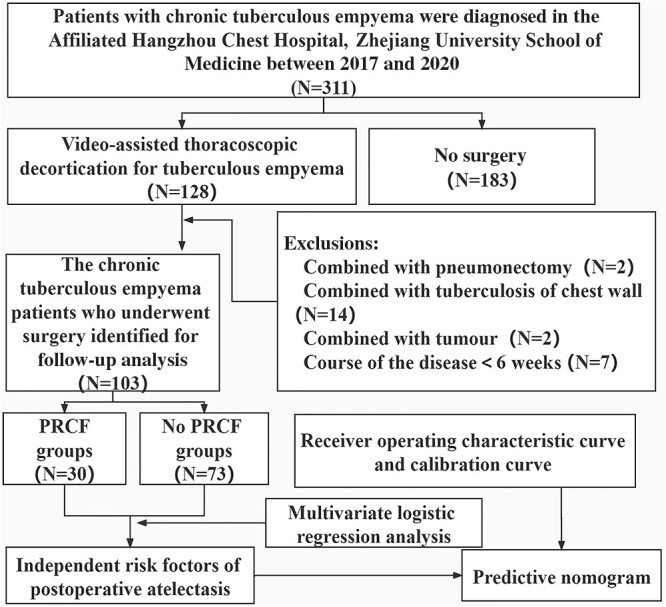
Flow chart of patients with chronic tuberculous empyema enrolled from our hospital from January 2017 to December 2020. Based on the inclusion and exclusion criteria, 103 patients were included in this study. PRCF: postoperative residual cavity formation.

Fig. 1 shows a flowchart of the study population. CTE was diagnosed in 311 patients; 128 patients with CTE who underwent video-assisted thoracoscopic decortication were included in this study. Of these, 128 were diagnosed by criterion 3, 121 by criterion 1 and 62 by criterion 2. The diagnosis of CTE was based on bacteriological detection, nucleic acid detection [10] and pathological examination. According to the inclusion and exclusion criteria, 103 patients were included in the final analysis of risk factors for residual cavity formation in patients with CTE. Residual cavity formation was observed in 30 cases (29.1%) and not observed in the remaining 73 cases (70.9%).

Baseline patient characteristics are shown in Table 1. No significant differences in sex, age, mean arterial pressure, heart rate, white blood cell count, creatinine, albumin, C-reactive protein, FEV1, LVEF, the duration of symptoms, treatment time of TB, lactate or pulmonary TB complications were detected between the groups. Compared with the no postoperative residual cavity formation group, the contact area between the lung and the empyema in the postoperative residual cavity formation group was larger (220.65 ± 86.44 cm^2^ vs 125.97 ± 69.53 cm^2^; *P* *<* 0.01). Patients with pleural calcification were more likely to develop postoperative residual cavity formation (60% [18/30] vs 12.3% [9/73]; *P* < 0.01). Furthermore, the pleurae in the postoperative residual cavity formation group were thicker than the pleurae in the no postoperative residual cavity formation group (11.6 ± 3.7 mm vs 7.1 ± 3.4 mm, *P* *<* 0.01).

**Table 1:** Baseline characteristics of patients in the postoperative residual cavity formation or no postoperative residual cavity formation groups

Variables	Postoperative residual cavity		P value
	Yes (N = 30)	No (N = 73)	
Sex			
Male	26 (86.7%)	46 (63%)	
Female	4(13.3%)	27(37%)	
Age, years	40 ± 15.9	33.7 ± 16.5	0.078
Clinical characteristics			
Cough	13（43.3%）	28（38.4%）	
Dyspnoea	11（36.7%）	22（30.2%）	
Chest pain	4（13.3%）	7（9.5%）	
Fever	2（6.7%）	16（21.9%）	
MAP, mmHg	87 ± 12	88.6 ± 10	0.499
Heart rate, beats/min	85.2 ± 10.7	82.1 ± 10.9	0.19
WBC, *10^9^/l	5.8 ± 1.9	5.3 ± 1.7	0.288
Creatinine, μmol/l	77.2 ± 10.2	77.2 ± 13.5	0.983
Albumin, g/l	39 ± 6.5	40.2 ± 5.6	0.363
CRP, cm H_2_o	26.4 ± 45.1	13.4 ± 24.6	0.144
FEV1, L	2 ± 0.6	2.2 ± 0.5	0.092
LVEF, %	63.2 ± 6.7	63.4 ± 4.5	0.799
Lactate, mmol/l	1.2 ± 0.3	1.3 ± 0.5	0.473
Treatment time of TB, months	8.4 ± 6.9	9.3 ± 5.3	0.509
Complicated with PTB	11（36.7%）	42（57.5%）	0.189
Area, cm²	220.65 ± 86.44	125.97 ± 69.53	<0.01
Calcification	18(60%)	9(12.3%)	<0.01
Thickness, mm	11.6 ± 3.7	7.1 ± 3.4	<0.01
Duration of symptoms, months	11.2 ± 15.2	17 ± 43	0.318

CRP: C-reactive protein; FEV1: forced expiratory volume in 1 s; LVEF: left ventricular ejection fraction (measured via echocardiogram); MAP: mean arterial pressure; PTB: pulmonary tuberculosis; TB: tuberculosis; WBC: white blood cells.

### Postoperative outcomes

The comparisons of the outcomes between the postoperative residual cavity formation group and the no postoperative residual cavity formation group are shown in Table 2. Compared with the no postoperative residual cavity formation group, duration of the operation was longer in the postoperative residual cavity formation group (3.53 ± 1.30 vs 2.98 ± 1.26, *P* = 0.049). Although the extubation time was comparable between the 2 groups, the postoperative hospital stay was shorter in the no postoperative residual cavity formation group (12.36 ± 5.43 vs 16.63 ± 7.58, *P* = 0.008). There were no in-hospital or 90-day deaths in either group. All included patients initially underwent VATS; 6 patients were converted to thoracotomy (4 (13.3%) in the postoperative residual cavity formation group and 2 (2.7%) in the no postoperative residual cavity formation group).

**Table 2:** Comparison of postoperative outcomes between the postoperative residual cavity formation and the no postoperative residual cavity formation groups

Characteristics	Postoperative residual cavity		p Value
	Yes (N = 30)	No (N = 73)	
Duration of the surgery, h	3.53 ± 1.3	2.98 ± 1.26	0.049
Extubation time, days	13.83 ± 5.26	12.29 ± 5.39	0.184
Postoperative hospital stay, days	16.63 ± 7.58	12.36 ± 5.43	0.008
In-hospital deaths	0	0	…
90-Day deaths	0	0	…
Conversion to thoracotomy	4 (13.3%)	2 (2.7%)	0.105

### Predictive factors associated with postoperative residual cavity formation

The variables in the univariable and multivariable analyses are summarized in Table 3. Univariable logistic regression analyses showed that the contact area of the lung and the empyema (*P* *<* 0.01), calcification (*P* *<* 0.01) and thickness (*P* *<* 0.01) were risk factors for postoperative residual cavity formation. The multivariable logistic analysis revealed that area (*P* = 0.001, OR 1.017, 95% CI 1.007–1.028), calcification (*P* = 0.004, OR 0.12, 95% CI 0.029–0.501) and thickness (*P* = 0.02, OR 1.315, 95% CI 1.045–1.654) were independent risk factors for postoperative residual cavity formation.

**Table 3:** Univariable and multivariable analyses of factors associated with the formation of a postoperative residual cavity

Variables	Univariable analysis			Multivariable analysis		
	P value	OR	95% CI	P value	OR	95% CI
Age	0.081	1.023	0.997-1.05	0.446	1.016	0.976-1.057
CRP	0.089	1.012	0.998-1.025	0.643	1.005	0.985-1.025
FEV1	0.095	0.482	0.204-1.137	0.536	0.658	0.174-2.481
Area	<0.01	1.015	1.008-1.022	0.001	1.017	1.007-1.028
Calcification	<0.01	0.108	0.039-0.294	0.004	0.12	0.029-0.501
Duration of the operation, h	0.054	1.394	0.995-1.953	0.255	0.674	0.341-1.329
Thickness	<0.01	1.448	1.222-1.716	0.02	1.315	1.045-1.654

CRP: C-reactive protein; FEV1: forced expiratory volume in 1 s.

### Establishment and validation of the nomogram model

**Figure 2:**
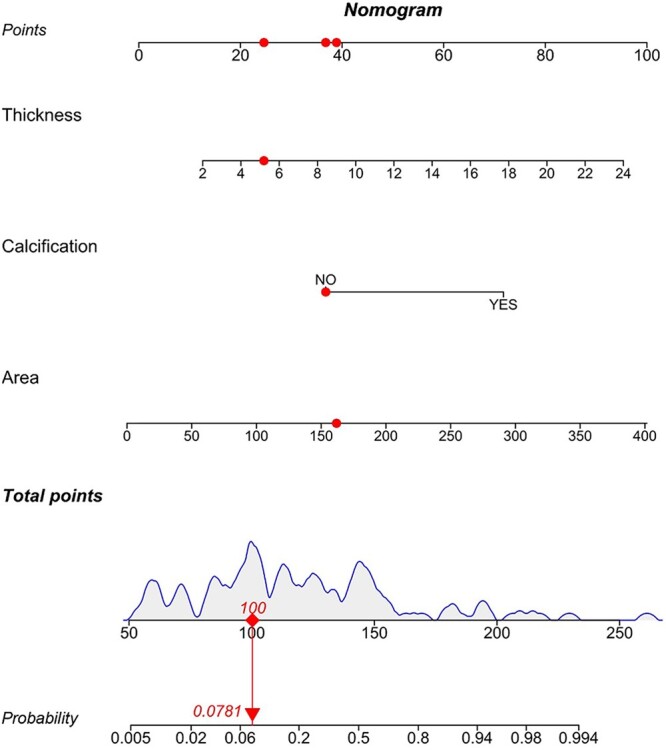
Nomogram for predicting the risk of residual cavity formation after video-assisted thoracoscopic decortication in chronic tuberculous empyema. Thickness: thickness of pleura; area:—the contact area between the lung and the empyema; calcification: pleural calcification.

**Figure 3:**
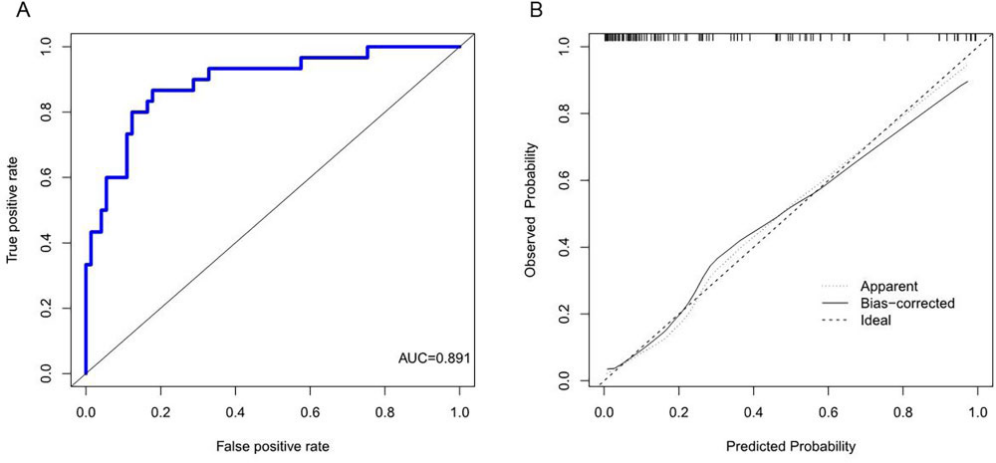
(A) Receiver operating characteristic curve of the nomogram. (**B)** Calibration curve for the nomogram.

Based on the independent risk factors, a nomogram was created (Fig. 2). In this nomogram, the contact area was the greatest predictor of postoperative residual cavity formation (100 points), followed by thickness (95 points) and calcification (72 points). The total score was 267. The score of each predictor is listed in Table 4. The corresponding postoperative residual cavity formation rate of each score is shown in Table 4. We used a 50% postoperative residual cavity formation rate as the classification cut-off, with a corresponding score of 144. We predicted that patients with scores >144 points would develop residual cavity formation, whereas patients with scores of 144 points or less would not develop residual cavity formation. The diagnostic performance of each predictive factor and model is summarized in Table 5. The ROC curve for the nomogram is presented in Fig. 3A; the area under the ROC curve was 0.891 (95% CI, 0.82–0.963). The sensitivity and specificity of the nomogram were 86.67% and 82.19%, respectively. The calibration curve [11] indicated good consistency between risk prediction using the model and actual risk (Fig. 3B).

**Table 4:** Scoring system for predicting postoperative residual cavity formation

Predictors	Scores
Calcification	
No	37
Yes	72
Thickness (mm)	
2	13
4	20
6	28
8	35
10	43
12	50
14	58
16	65
18	73
20	80
22	88
24	95
Area (cm^2^)	
0	−2
50	10
100	23
150	36
200	49
250	61
300	74
350	87
400	100
Total	267
Atelectasis rate	
0.00028	0
0.00481	50
0.07699	100
0.59010	150
0.96130	200
0.99770	250
0.99990	300

Thickness: thickness of pleura; area: the contact area between lung and empyema; calcification: pleural calcification.

**Table 5:** Diagnostic performance of each predictive factor and overall model

Variables	Cut-off	AUC	OR (95% CI)	P Value	Sensitivity	Specificity
Area (cm^2^)	186.46	0.795	0.703-0.888	<0.01	63.33	86.3
Thickness	8.75	0.822	0.742-0.903	<0.01	86.7	71.2
Calcification						
YES		0.722	0.604-0.84	<0.01	60	87.7
NO		Reference				
Nomogram		0.891	0.82-0.963	<0.01	86.67	82.19

Thickness: thickness of pleura.

Area: the contact area between the lung and the empyema.

Calcification: calcification of the pleura.

AUC: area under curve; OR: odd ratio.

## DISCUSSION

CTE can seriously affect the cardiac and pulmonary functions of patients when left untreated. CTE causes chest deformity, intercostal space narrowing, haemoptysis, and bronchopleural fistulae. The objective of CTE treatment is to eliminate infection and the pus cavity so that pulmonary function can be regained [12–15]. At present, the preferred treatment for CTE is decortication. The General Thoracic Surgery Database of the Society of Thoracic Surgeons revealed that the in-hospital complications and mortality rates after decortication were approximately 39% and 3.1% [16], respectively. Decortication via VATS is both feasible and safe [17, 18]. Despite the effectiveness in treating CTE, decortication may cause postoperative complications, such as postoperative residual cavity formation. Postoperative residual cavity formation is a common finding on plain chest radiographs and can usually be seen with CT. Mueller *et al.* reported that the incidence of residual cavity formation after surgery for the management of TB was between 21% and 33% [19]. In agreement with this study, we detected an incidence of residual cavity formation after decortication of 29.1%.

Most residual cavities are absorbed within 4 weeks postoperatively, and the incidence of residual cavities is reduced over time [20]. Approximately 14% of patients who underwent decortication develop complex cavities that require redrainage or reoperation. The minor traumatic techniques of thoracoplasty and myoplasty are salvage options for resolving treatment-refractory complicated residual pleural spaces [21].

Melloni and his colleagues [22] conducted a prospective study on the outcome of decortication for CTE in Italy. Their study demonstrated that symptom duration and duration of conservative treatment were associated with complications, including the formation of a residual cavity. In contrast, in our study, the duration of symptoms was not an independent risk factor for the formation of residual cavities. However, most patients with CTE are asymptomatic, and pinpointing the actual time of onset is difficult, causing a statistical bias.

According to our univariable analyses, the postoperative formation of residual cavities was associated with calcification, the contact area between the lung and the empyema, the duration of the operation, the thickness of the pleurae, age and FEV1. However, the multivariable analysis demonstrated that only calcification, contact area between the lung and the empyema and the thickness of the pleurae are independent risk factors for the postoperative formation of residual cavities. The risk factors provide valuable guidance for preoperative preparation and risk evaluation. The area of contact between the lung and empyema is the area that needs to be dissected during the operation. Bleeding, air leakage and oedema can occur over the dissection surface during dissection of the visceral pleural. Thus, a larger anatomical area of dissection causes a greater risk of lung injury. Injury to the lung surface can lead to atelectasis and postoperative cavity formation. The degree of pleural adhesion is important for the prognosis of decortication. Calcification of the pleura suggests severe pleural adhesions that are difficult to dissect. For pleurae that are difficult to dissect, a modified pleural decortication, which preserves part of the thickened parietal pleura, is an option. However, we believe that decortication of the visceral pleura can promote lung re-expansion, increase thoracic compliance and reduce the formation of residual cavities to improve lung function.

In this study, we developed a nomogram for predicting residual cavity formation after thoracoscopic decortication in CTE. This nomogram incorporated 3 variables, including contact area between the lung and the empyema, pleural calcification and thickness of the pleura. These 3 variables were obtained from observing CT images, which were all simple and easy to measure clinically. Among the variables constituting nomograms, many studies have shown that the duration of antituberculosis therapy is an independent risk factor affecting the prognosis of the empyema [23]. Unfortunately, the duration of antituberculosis therapy was not a significant risk factor in this nomogram. It might be due to the small data set. In the future, we will increase the data set to study the relevance between the duration of antituberculosis therapy and residual cavity formation after thoracoscopic decortication in CTE. After this analysis, further research is needed to determine if the duration of antituberculosis therapy is a reasonable component of the nomogram. Because of the small data set, we were unable to conduct internal cross-validation or external validation. However, the nomogram has an AUC value of 0.891 on internal validation. The sensitivity and specificity of the nomogram were 86.67% and 82.19%, respectively. The calibration curve indicated good consistency between risk prediction using the model and actual risk. Given these results, this preliminary model is expected to facilitate preoperative counseling and to be used to establish customized perioperative strategies.

This study has some limitations. First, to evaluate the model, we used an internal validation method that may have overestimated the diagnostic accuracy of the model. Therefore, prospective external validation of the model is needed. Second, the data were collected from a single centre; multiagency, large-sample research is warranted. Third, the experience of the operating radiologist is difficult to quantify but may be a significant factor in the prediction of residual cavity formation after surgery.

## CONCLUSIONS

Our study suggests that the contact area between the lung and the empyema, the thickness of the pleurae and pleural calcification are independent risk factors for postoperative residual cavity formation. Furthermore, the predictive nomogram, which combines imaging features and clinical factors, is expected to be a convenient decision-making tool to prevent residual cavity formation and make appropriate surgical decisions.

### Data availability statement:

The data underlying this article will be shared on reasonable request to the corresponding author.

### Funding

No funding.

### Conflict of interest

The authors declare that there is no conflict of interest.

### Author contributions

Dr. Pengfei Zhu and Dr. Xudong Xu contributed to conceptualization; Dr. Pengfei Zhu, Dr. Likui Fang, and Dr. Wenfeng Yu contributed to formal analysis and writing—review & editing; Dr. Pengfei Zhu, Dr. Xiaowei QIU, Dr. Guocan Yu, and Dr. Xin Yang contributed to investigation, data curation, and formal analysis. Dr. Bo Ye, Dr. Fangmin Zhong, and Dr. Xudong Xu contributed to writing—review & editing. All authors have approved the final draft of the manuscript.

### Consent for publication

Not applicable.

## Supplementary Material

ivac011_Supplementary_Data

## Abbreviations

AUC: area under the curve
CTE: chronic tuberculous empyema
CT: computed tomography
CI: confidence interval
FEV1: forced expiratory volume in 1s
LVEF: left ventricular ejection fraction
OR: odds ratio
ROC: receiver operating characteristic curve
TB: tuberculosis
VATS: video-assisted thoracoscopic surgery
