# Supplementary figures and images for: A nomogram to predict residual cavity formation after thoracoscopic decortication in chronic tuberculous empyema
Source: Interact Cardiovasc Thorac Surg. 2022 Feb 11;34(5):760–7. doi: 10.1093/icvts/ivac011 (PMC9070475; doi:10.1093/icvts/ivac011)

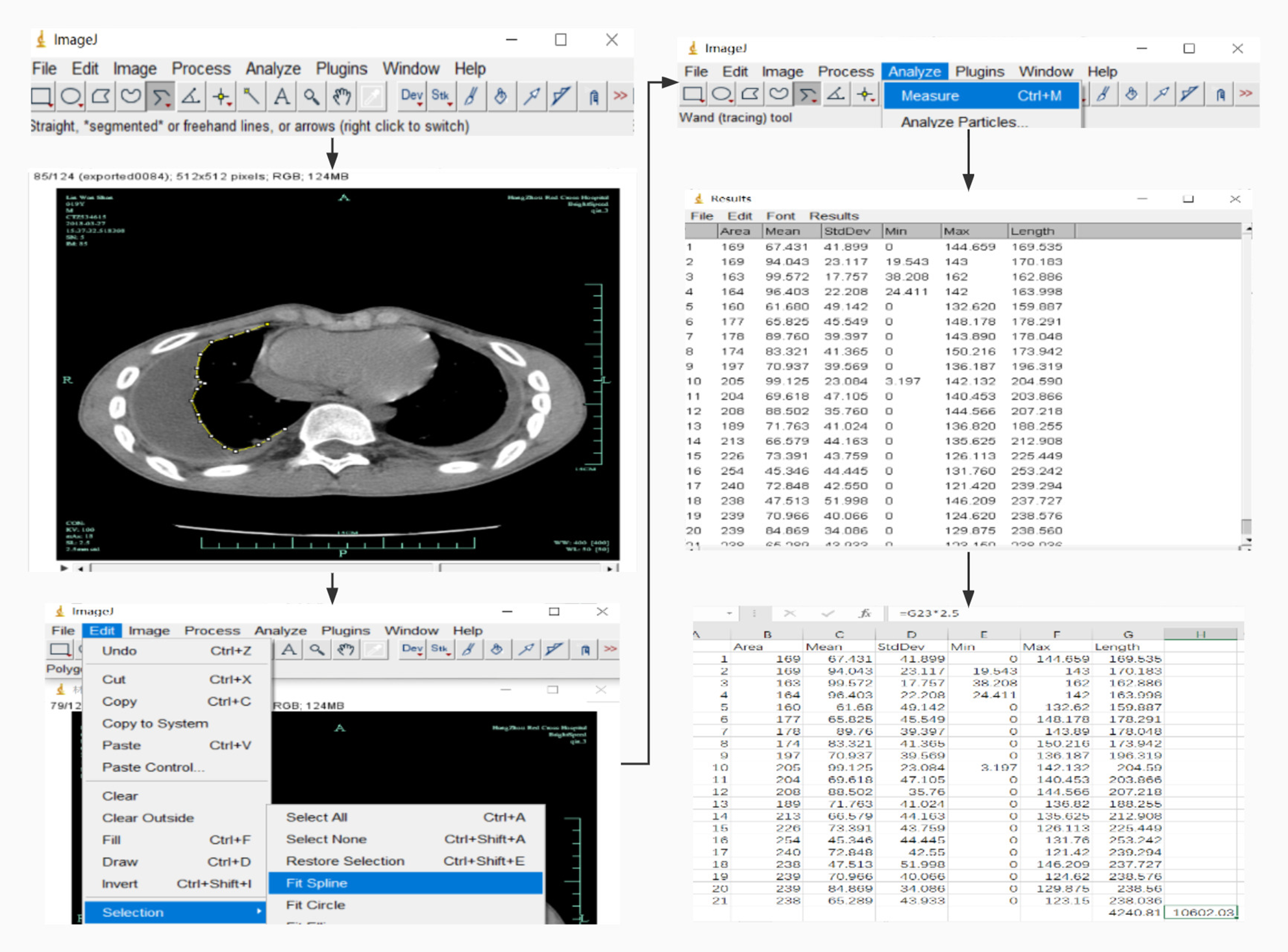

Supplement: ivac011_Supplementary_Data [file ivac011_supplementary_data.zip › ivac011_Supplementary_Data.tif]
